# Influence of Age on Skeletal Muscle Hypertrophy and Atrophy Signaling: Established Paradigms and Unexpected Links

**DOI:** 10.3390/genes12050688

**Published:** 2021-05-03

**Authors:** Eun-Joo Lee, Ronald L. Neppl

**Affiliations:** Department of Orthopaedic Surgery, Brigham and Women’s Hospital, Harvard Medical School, Boston, MA 02115, USA; elee59@bwh.harvard.edu

**Keywords:** Akt signaling, muscle atrophy, muscle hypertrophy, IGF-1/PI3K signaling, aging muscle, RNA-binding proteins

## Abstract

Skeletal muscle atrophy in an inevitable occurrence with advancing age, and a consequence of disease including cancer. Muscle atrophy in the elderly is managed by a regimen of resistance exercise and increased protein intake. Understanding the signaling that regulates muscle mass may identify potential therapeutic targets for the prevention and reversal of muscle atrophy in metabolic and neuromuscular diseases. This review covers the major anabolic and catabolic pathways that regulate skeletal muscle mass, with a focus on recent progress and potential new players.

## 1. Introduction

Biological aging is broadly defined as the time-dependent loss of functionality and robustness, and is associated with increased rates of cancer [1,2], cardiovascular and neurodegenerative disease [2,3,4], diabetes or dysglycemia [5], insulin and anabolic resistance [6,7,8]. Many of these disorders and diseases are often accompanied by, and contribute to, muscle weakness and sarcopenia, exacerbating the slow gradual loss of muscle mass and strength that typically begins in the fourth decade of life [9,10,11]. In healthy young individuals, lean muscle accounts for 38–54% and 28–39% of total body mass, in men and women, respectively. These ranges are quite broad and are dependent upon multiple factors including physical activity level, overall health, genetic makeup, and nutritional input. Skeletal muscle is comprised of a heterogenous mix of specialized myofibers that differ in their physiological, metabolic, and biochemical attributes. On the two ends of this spectrum are slow oxidative fibers, comprised of type I myosin heavy chain proteins (MHC), and the fast-glycolytic fibers, comprised of types IIa, IIx, and IIb (in rodents) MHC proteins. With advancing age, skeletal muscle loses its responsiveness to anabolic signals, which are secreted in ever diminishing levels [8,12,13,14]. Importantly, fast-glycolytic fibers atrophy at a greater rate than oxidative fibers with advancing age [15,16,17], contributing significantly to the loss of muscle power that begins its decline from approximately age 40 onwards [18]. It is this loss of muscle power that is related to the increased number of falls among the elderly [18,19].

The cellular and molecular mechanisms linking biological aging with the loss of muscle mass, strength, and functionality are complex. In its simplest form, the maintenance of muscle mass, and strength, is due to the balance of anabolic and catabolic processes. Stem cell exhaustion, cellular senescence, altered intercellular communication, losses of proteostasis and genome stability, telomere attrition, mitochondrial dysfunction, epigenetic alterations, and dysregulation of gene expression and alternative splicing are hallmarks of, and causal for, the age-associated loss of cellular and biological robustness [20,21,22,23]. In this review, we identify and discuss the cellular and molecular mechanisms responsible for modulating skeletal muscle mass and strength, and its influence on the non-myocyte contributions to skeletal muscle physiology that become dysregulated with age. Herein we give high priority to in vivo and clinical studies identifying the effects of age on the linkages between gene expression and signaling pathways with muscle function. Finally, we would like to sincerely apologize to our many outstanding colleagues, whose work may have been inadvertently overlooked or not thoroughly discussed due to space constraints.

## 2. PI3K/Akt/mTOR Signaling in Muscle

**Figure 1:**
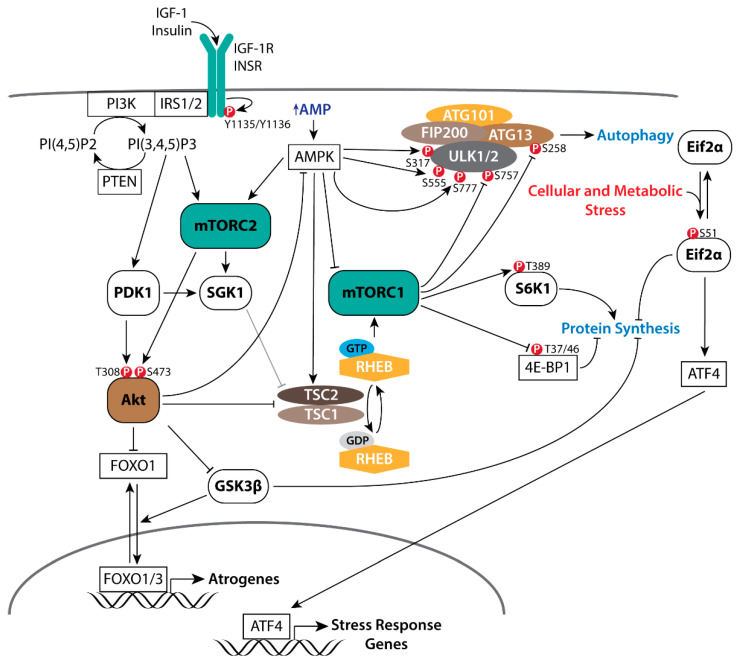
Signaling pathways downstream of insulin/IGF-1 receptor activation and crosstalk with catabolic signaling pathways. Signaling pathways observed in multiple cells lines and tissues in vivo (black lines). Signaling pathways observed in cancer cells resistant to PI3K inhibitors (grey lines).

Insulin, and insulin-like growth factor 1 (IGF-1) signaling (IIS) is ubiquitous to multicellular animals, connecting nutrient availability to development, longevity, and growth in response to hypothalamic-pituitary growth signals [24]. Activation of the insulin/IGF-1 receptors leads to the phospho-activation of phosphoinositide 3-kinase (PI3K), and the subsequent activation of Akt (Figure 1), a key integrator of intra- and intercellular signals known to promote cell survival, glucose metabolism, and anabolic growth processes [25,26,27,28,29,30,31,32]. Importantly, IIS has evolutionarily conserved pleiotropic effects that are age/developmental stage and gender dependent. Ames, Snell, and *GHRH* (*lit*/*lit*) dwarf mice have reduced growth hormone (GH) signaling and reduced serum IGF-1 levels, but have up to a 70% extension of their lifespans [33,34,35,36,37,38]. Heterozygous deletion of the IGF-1 receptor gene (*Igf1r*) have a modest ~6–8% reduction in bodyweight by 7 weeks of age, but live up to 25% longer [39,40]. However, this lifespan extension was limited to females and modulated by genetic background [39,41,42]. Consistent with the influence of gender, 18-month-old female mice treated with a selective IGF-1R antagonist for 6 months, but not male mice, showed significant improvements in exercise tolerance, grip strength, and motor coordination [43]. In contrast, transgenic expression of GH in mice induces accelerated growth, hyperinsulinemia despite euglycemia, increased adult body weight and plasma IGF-1 levels, and a nearly 50% reduction in lifespan [44,45,46,47].

PI3K/Akt/mTOR signaling is a key regulator of glycolytic muscle homeostasis, hypertrophic growth, and metabolism through its activation of mTOR-dependent anabolic processes while simultaneously inhibiting both autophagy and Foxo1/3 catabolic processes [48,49]. Skeletal muscle-specific transgenic overexpression of Akt selectively promotes muscle hypertrophy [50]. Glycolytic muscle specific transgenic overexpression of Akt similarly induces glycolytic muscle hypertrophy, promoting weight loss and insulin sensitivity in obese [51] and aged mice [52]. Functional overload [53], IGF-1 stimulation [54,55], and skeletal muscle specific transgenic overexpression of IGF-1 [56] positively regulate PI3K/Akt/mTOR signaling in muscle and promote hypertrophic growth. The phospho-activation of Akt is initiated by a series of intracellular signaling events arising from receptor tyrosine kinases (RTKs) including the IGF-1 (IGF-1R) and insulin (Insr) receptors, and G-protein-coupled receptors (GPCR), which signal through scaffolding proteins (e.g., IRS1/2, Grb2/10) to activate PI3K. Active PI3K produces phosphatidylinositol (3,4,5)-trisphosphate (PIP3), leading to the phospho-activation of Akt at T308 by PDK1. Maximal activation of Akt requires phosphorylation at the S473 site, which is directly phosphorylated by mTORC2 [26,29]. Comprised of core components mTor, Rictor, SIN1, and mLST8, mTORC2 functions primarily as an effector of insulin/PI3K activity [57]. Though the regulation of mTORC2 is uncertain, a growing body of evidence would suggest that mTORC2 activity is positively modulated through its association with ribosomes, the mitochondrial-associated ER membrane, and lysosomes [58,59,60,61,62,63,64,65,66]. Physiological regulation of Akt activation is achieved, in part, by the PIP3 phosphatase PTEN and members of inositol polyphosphate family of Type IA phosphatases (e.g., Inpp4a, Inpp5a) [57,67,68]. Though critical in limiting Akt activation, PTEN is now being recognized for its role in modulating insulin sensitivity and energy expenditure [69]; mice with chronic diabetes or insulin resistance have increased PTEN protein expression [70], while PTEN transgenic mice have decreased muscle mass coincident with increased energy expenditures [71].

Activated Akt acts directly on multiple targets to modulate cellular growth and metabolic processes (reviewed in [29]). Akt phosphorylates TSC2 to repress the GAP activity of the TSC1/TSC2 complex toward RHEB (Figure 1), maintaining RHEB in the GTP-bound state (RHEB•GTP) to activate mTORC1 [72,73,74]. In cancer cells resistant to PI3K inhibitors, SGK1 similarly phosphorylates TSC2 (Figure 1) to activate mTORC1 [75] in response to direct phosphorylation at S422 by mTORC2 [76,77] or PDK1 [78,79]. In addition, direct phosphorylation of PRAS40 at T246 by Akt blocks its inhibition of mTORC1 [80,81,82]. Although PRAS40 can inhibit RHEB•GTP activation of mTORC1 [80], insulin signaling in *Tsc2* null MEFs expressing a phospho-deficient form of TSC2 failed to induce mTORC1 signaling despite the phosphorylation of PRAS40 at T246 [83], suggesting that TSC-RHEB regulation of mTORC1 activation predominates. GSK3 is a constitutively active Ser/Thr protein kinase with a large set of functionally diverse direct targets, the majority of which are functionally inhibited or degraded following GSK3-directred phosphorylation [84,85]. FoxO transcription factors are associated with longevity in humans [86], in part, through modulating the expression of genes involved in apoptosis, glucose metabolism, cell cycle, DNA damage repair, and proteolysis [87]. FoxO proteins are exported from the nucleus and sequestered in the cytoplasm when phosphorylated at their N-terminus and NLS sequences (T24 and S256 on FoxO1) [88]. Akt phosphorylates both GSK3 [89,90] and FoxO transcription factors [91,92] to inhibit their respective kinase and transactivating activities.

The mTOR-containing complex 1 (mTORC1) is a major effector of activated Akt, stimulating the synthesis of proteins, lipids, and nucleotides, while inhibiting protein degradation pathways [57]. Recruitment of the small ribosomal subunit to mRNA is dependent upon binding of the mRNA 5′ cap structure by the eukaryotic translation initiation factor 4F (eIF4F) complex. Comprised of the initiation factors eIF4E, eIF4G, and eiF4A, the eIF4F complex is critical for cap-dependent protein synthesis [93,94]. mTORC1 directly phosphorylates the eIF4E inhibitor 4E-BP1 at T37/46 to promote assembly of the eIF4F complex on the 5′ cap of mRNA [57,95,96]. In addition, mTORC1 phosphorylates S6K1 at T389 [97,98], activating its kinase activity toward the 40S ribosomal protein S6 (rpS6) [99], eIF4B [100], and the eIF4B inhibitor PDCD4 [101] to stimulate protein synthesis. Phosphorylation of eIF4B at S422 by S6K1/2 stimulates eIF4F activity by potentially promoting the ATPase, RNA-binding, and RNA-helicase activities of eIF4A [93,100,102]. Akt/mTORC1 signaling promotes de novo lipid synthesis through promoting the nuclear accumulation of SREBP to initiate the transcription of metabolic genes involved in fatty acid synthesis [103,104]. Further, pyrimidine synthesis is stimulated through S6K1 phosphorylation of CAD [105], while mTORC1-dependent purine synthesis is through activation of the mitochondrial tetrahydrofolate cycle, in part, through induction of *MTHDF2* by eIF2α-independent activation of ATF4 [106]. In response to cellular stress (e.g., amino acid insufficiency, unfolded protein response of the endoplasmic reticulum) Eif2α is phosphorylated at S51 (Figure 1) to both inhibit cap-dependent mRNA translation and to initiate the transcription of stress response genes including *FGF21*, *SQSTM1*, *ATG3*, *ATG12,* and Sestrin2 (*Sesn2*) via ATF4 [107,108,109]. Lastly, autophagy is an essential process for the maintenance of cellular homeostasis that may be further induced in response to cellular stress to reduce organelles and macromolecules into amino acids, nucleosides, fatty acids and sugars in support of cellular growth and survival [110]. Under growth conditions, active mTORC1 negatively regulates autophagy (Figure 1) through its direct phosphorylation of ULK1 at S757 [111] and Atg13 at S258 [112,113].

Nutrient and energy availability is a critical regulator of mTORC1. Increases and decreases in amino acids leucine and arginine positively and negatively modulate mTORC1 kinase activity via CASTOR1, Sestrin2, and SLC38A9 [114,115,116,117,118], thus providing a critical feedback mechanism to modulate mTORC1 activity in response to nutrient availability. Similarly, under low ATP conditions, AMPK may negatively modulate mTORC1 kinase activity (Figure 1) through phosphorylation of the mTORC1-specific constituent scaffolding protein Raptor [119] or through the phospho-activation of TSC2 [120,121]. Further, AMPK phosphorylates mTOR at S1261 within mTORC2, while AICAR-induced AMPK activation both increases and decreases mTOR autophosphorylation at S2481 when associated with Rictor and Raptor, respectively [62]. Although the mTOR S1261 phosphorylation was found to be dispensable for the AMPK-dependent increase in mTORC2 catalytic activity, AMPK-mTORC2 signaling was found to promote cell survival following glucose withdrawal.

Multiple human studies have indicated significantly reduced rates of mixed, myofibrillar and/or mitochondrial protein synthesis in the muscles of elderly subjects [122,123,124,125,126,127,128,129]. However, other more recent human studies show little or no difference in basal muscle protein synthesis rates between the young and elderly [8,130,131,132,133,134,135,136]. While differences in physical activity level, genetic makeup, and nutritional input clearly contribute to these earlier observations, the previously reported >30% reductions in basal muscle protein synthesis rates [122,123,124,125,126,127,128,129] may not be reflective of normal physiological aging. Differences in muscle biopsy locations, analysis methods accounting for myofibrillar and total protein synthesis rates, and/or subject screening for average physical activity levels, nutritional intake, and overall health may similarly contribute. Additional studies with larger cohorts utilizing methods with improved sensitivity to protein synthesis while incorporating measures of protein degradation (e.g., proteosomal and autophagic flux) are warranted.

Resistance training increases muscle protein synthesis in both young and elderly men and women [122,124,126,128,137,138,139,140]. Exogenous and post-prandial amino acids increase muscle protein synthesis in both young and elderly men and women at baseline [8,133,141,142,143], while providing an additive effect post exercise [144]. However, the efficacy of both exercise and amino acids on stimulating muscle protein synthesis is blunted in the elderly [8,139,145], a phenomenon referred to as anabolic resistance. This occurs despite increased plasma concentrations of leucine in elderly subjects, as compared to young, following both exercise and ingestion of essential amino acids [8]. Further, elderly individuals, as compared to young controls, have blunted inductions of Akt phosphorylation at T308 and S473, S6K1 phosphorylation at T389, and 4E-BP1 phosphorylation at T37/46 after both exercise alone, and amino acid supplementation after exercise [145,146], strongly suggesting that PI3K/Akt/mTOR signaling dysregulation mechanistically contributes to anabolic resistance.

In vivo mechanistic studies in animal models add further support to this notion. Reductions in Akt phosphorylation at S473 and S6K1 phosphorylation at T389 are observed in the gastrocnemius of old mice as compared to the young controls [147]. This study further employed targeted transcriptomic and metabolomic analyses, revealing dysregulation of fatty acid and glucose metabolic processes in old mice. In contrast, significant increases in basal Akt phosphorylation at S473 [148] and S6K1 phosphorylation at T389 [149], are observed in the muscles of 33- and 24-month old rats, respectively. RpS6 phosphorylation at S240/244 is similarly increased in the 24-month old rats [149], indicating activation of a subset of anabolic signaling branchpoints. It should be noted that the increase in Akt S473 phosphorylation at 33-months was observed in the slow oxidative soleus, rather than fast glycolytic muscles predominantly examined for Akt signaling. Interestingly, the slow oxidative myofibers of the soleus, similar to fast glycolytic myofibers of the gastrocnemius of rats, positively modulate Akt phosphorylation at S473 following sciatic nerve stimulation [150] and 30 min of treadmill exercise [151]. Importantly, glycogen content in the soleus (65% reduction) was reduced to a greater extent than in the red (47% reduction) and white (23% reduction) gastrocnemius following treadmill exercise [151], suggesting that insulin-stimulated PI3K/Akt-regulated glucose uptake and glycogen storage is similarly activated in oxidative muscles in response to exercise. However, the extent to which Akt signaling modulates oxidative muscle metabolism through its influence on PGC-1α transcriptional activity in vivo [152], is uncertain and warrants future investigation.

The percentages of histologically abnormal myofibers, and myofibers staining positive for both phosphorylated rpS6 and active caspase-3 are increased in the muscles of both old mice and humans [153]. Counterintuitively, muscle-specific activation of mTORC1 (*Tsc1* knockout) in mice induces progressive myofiber atrophy, phenocopying many of the histological features of aged muscles including increases in both myofiber vacuolation and the number of caspase-3 positive myofibers [153,154]. Similarly, pharmacological inhibition of mTORC1 reduced myofiber atrophy and the expression of both Atrogin-1 and MuRF1 in a mouse model of cancer cachexia [155]. In contrast, short-term activation of mTORC1 (TSC2 knockdown) specifically in skeletal muscle induced myofiber hypertrophy and greatly increased hypertrophy on recovery from transient denervation [156]. Consistent with these observations, muscle-specific inhibition of mTORC1 (*Raptor* knockout) in mice induced a leftward shift in the myofiber size distribution curve and histological features including centralized nuclei and vacuoles, and myofiber degeneration [157]. These apparently discordant observations with respect to myofiber size may more accurately be rationalized through the effects of Akt/mTORC1 signaling on metabolism, rather than simply the balance of protein synthesis and protein degradation. Short-term activation of Akt induces myofiber hypertrophy [51,52] in parallel with a metabolic shift from glycolysis and oxidative phosphorylation toward the accumulation of branched chain amino acids and enhancement of the pentose phosphate pathway (PPP) [158]. Importantly, PPP produces ribose 5-phosphate and NADPH which are critical for the production of nucleotides, fatty acids, sterols, non-essential amino acids, and cellular antioxidant defenses via reduced glutathione [159]. These observations would suggest that the hypertrophic effects of short-term Akt/mTORC1 signaling become deleterious when prolonged, prioritizing the synthesis of anabolic precursors rather than energy production, and leading to cellular and metabolic stress. Increased expression of pro-inflammatory cytokines (e.g., *Ccl2*, *Ccl3*, *Il10*) and metabolic stress indicators (e.g., *FGF21*, *GDF3*) in the hypertrophic muscles of Akt transgenic mice [158], and in atrophied muscles in which Akt is observed to be constitutively active [160], further support this notion.

Treatment with rapamycin both extends the lifespan of mice and fruit flies [161,162,163] and blocks resistance exercise-mediated increases in protein synthesis in humans [164,165]. Akt phosphorylation at both T308 and S473 are increased in muscle following a 16-h fast in 24- and 30-month old male mice with respect to 6-month old controls [166]. Male mice in this study also had significant increases in S6 phosphorylation at S240/244, whereas in contrast, aged female mice only showed an increase in Akt S473 phosphorylation without the increase in S6 phosphorylation after fasting [166]. Though suggestive of positive regulation of Akt via mTORC2 following a fast in aged mice, interpretation of these observations may be confounded by multiple variables including fasting duration, species, gender, systemic and muscle metabolic flux, as well as housing and dietary conditions throughout the lifespan of experimental animals. It is important to note that fasting in young healthy mice, in which systemic control of blood sugar and insulin is physiologically normal, induces the positive regulation of proteolytic processes through increased FoxO transcriptional activity [167] and AMPK induction of autophagy through phosphorylation of ULK1 at Serines 317, 555, and/or 777 (Figure 1) [111,168,169] while inhibiting protein synthesis [49,170]. Consistent with this, pharmacological inhibition of mTORC1 restored muscle histological and functional parameters in *Tsc1* mutant mice [154,171] and in aged rats [149], in part, through restoration of autophagy.

## 3. Muscle Proteolytic Processes and Negative Regulation of Anabolic Processes

Aging and aging-related diseases are linked to impaired proteome integrity or proteostasis [172,173]. Acute and gradual increases in oxidative damage negatively impact protein stability and enzymatic function [174,175,176,177]; 40–50% of all proteins in old organisms are estimated to have some form of oxidative damage [178]. Similarly, the accumulation of misfolded proteins is known to contribute to the development of age-associated pathologies including Alzheimer’s disease, Huntington’s disease, and sporadic inclusion-body myopathy [172,173,179,180].

Cells have evolved multiple protein degradation processes to maintain proteome integrity or proteostasis in response to endogenous and exogenous stresses that negatively impact the protein functionality due to damage or misfolding. Approximately 60–80% of protein degradation in growing mammalian cells occurs through 26S proteasome catalysis [181,182,183,184]. Specificity is achieved through the selective polyubiquitylation of substrates by specific E3 ubiquitin ligases. While the proteasome can degrade some non-ubiquitinated proteins, ubiquitination may function independently of the proteasome by selectively directing proteins (typically membrane proteins) to the lysosome for proteolysis [184]. Similarly, autophagy reduces organelles and macromolecules into amino acids, nucleosides, fatty acids, and sugars in support of cellular growth and survival [110]. The core autophagy-related proteins essential for formation of the autophagosome formation and delivery of autophagic cargo to the lysosome may be grouped by functionally: (1) initiation and phagophore nucleation proteins ULK1, ULK2, ATG1, FIP200, ATG13, VPS34, ATG9, Beclin, (2) phagophore expansion proteins ATG3, ATG4, ATG7, ATG9, ATG10, ATG12, (3) cargo sequestration and membrane sealing proteins ubiquitin, cardiolipin, p62/SQSTM1, PE-conjugated LC3s and GABARAP, and (4) autophagosome maturation and lysosome fusion proteins ATG4, ATG14, VCP, and PE-conjugated LC3s and GABARAP [185,186].

In humans and animal models, these proteolytic processes become dysregulated with pathologies associated with muscle atrophy including cancer cachexia, chronic renal failure, diabetes, denervation, and age [187,188,189]. Atrophying muscles in old [190] and in young thyroidectomized and tumor bearing rats [191] have increased levels of protein polyubiquitylation. Consistent with the experimental models of disuse muscle atrophy in mice [192], increased levels of protein polyubiquitylation are observed in the muscles of young human volunteers following an extended 20-day bed rest [193]. Gene expression analyses during the early stages of muscle wasting due to denervation, disuse, diabetes, cachexia, and fasting have identified a common set of genes, referred to as atrogenes, whose expression is coordinately induced or repressed under conditions of muscle atrophy or growth. Atrogenes whose expression is increased in atrophying muscles include ubiquitin, core components of the 26S proteosome, eIF4E inhibitor 4E-BP1 (*Eif4ebp1*), transcription factors *Atf4*, *Foxo1*, and *Foxo3* [194,195], the muscle specific E3 ubiquitin ligases MurF1 (*Trim63*) and Atrogin-1/MAFbx (*Fbxo32*) [196,197], and autophagosome components LC3 (*Map1lc3a*) and *Gabarap* [194,198,199]. Importantly, animal models indicate that both insufficient [200,201,202,203] and excessive [198,199,204] proteolysis, both proteasomal and autophagic, negatively impact the muscle mass and functionality, suggesting an inflection point between a level of proteolysis that supports muscle homeostasis and that which promotes muscle wasting.

**Figure 2:**
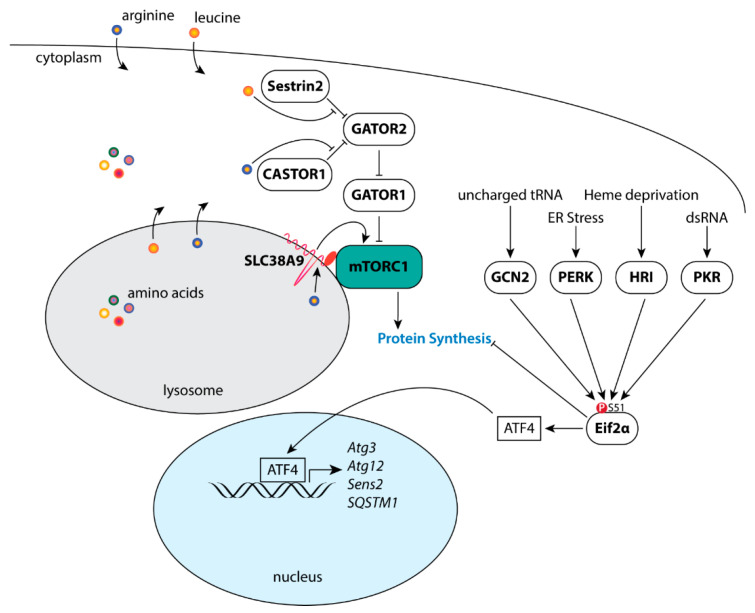
Nutrient sensing pathways modulate protein synthesis. Cells respond to cellular stress signals to both inhibit protein synthesis and increase the expression of genes to alleviate cellular stress.

Autophagy and ATF4 are both positively regulated in response to cellular stress (Figure 2) and major effectors of the integrated stress response (ISR). The ISR is a multifaceted signaling pathway that concurrently represses cap-dependent protein synthesis via Eif2α while inducing the translation of select mRNAs which contain short inhibitory upstream open reading frames (uORFs) in their 5′-untranslated regions including *ATF4*, *DDIT3* (CHOP), and *PPP1R15A* (GADD34) [205]. The Eif2α kinases PERK, PKR, and HRI converge to phosphorylate Eif2α at S51 in response to the unfolded protein response of the endoplasmic reticulum (UPR^ER^) [206,207], dsRNA [208,209], and heme deprivation [210,211], respectively. In addition, amino acid sensing plays a key role in both activating the ISR as well as inhibiting mTORC1 (Figure 2). Reduced levels of amino acids lead to both an increase in the levels of uncharged tRNAs thereby activating GCN2 kinase activity toward Eif2α [212], and a concurrent reduction in mTORC1 activity via Sestrin2 [115], CASTOR1 [213] and SLC38A9 [214,215,216].

FoxO transcription factors are positive regulators of Atrogin-1, MuRF1, LC3, GABARAPs, ULK2, ATG12, and Beclin [198,199,217,218], thus indicating their central role in promoting proteolytic processes. Transgenic overexpression of *Foxo1* specifically in skeletal muscle results in mice with decreases in muscle mass, functionality, and sarcomeric protein expression [219], while muscle specific *Foxo1* knockout mice were largely protected from nephrectomy and dexamethasone-induced muscle atrophy [220]. Muscle-specific genetic deletion of all three muscle Foxo transcription factors (*Foxo1*, *Foxo3*, and *Foxo4*) abolished both fasting [221] and insulin-deficient diabetes [222] induced muscle loss and weakness. Consistent with these observations, heterozygous deletion of either Atrogin-1 or MuRF1 results in mice that are partially protected from denervation-associated muscle atrophy [196]. Similarly, overexpression of a constitutively active form of FoxO3 in tibialis anterior muscles markedly increased the autophagosome formation and myofiber atrophy while siRNAs targeting *Foxo3* and overexpression of a dominant negative form of FoxO3 blocked fasting-induced autophagy [198].

Proteins may also be cleaved by members of the caspase family of proteases which are known to play key roles in regulating apoptosis, necrosis, and inflammation [223,224]. Perhaps best known for its role in the activation of IL-1β [224], caspase-1 enzymatic activity is observed to be significantly increased in the muscles of 24-month old mice as compared to 10-month old controls [225]. Interestingly, genetic deletion of *Nlrp3* blunted both the age-associated changes in muscle mass and caspase-1 activity, respectively [225], thus demonstrating a mechanistic link between the NLRP3 inflammasome and age-related muscle atrophy. The muscles of 24-month old rats show no statistical differences in either cytosolic cytochrome *c* levels or cytosolic caspase-3 activity from 6-month old controls [226], suggesting that mitochondrial-mediated caspase activation is not present at this timepoint. Further, unlike in liver homogenates, cytochrome *c* stimulation of muscle homogenates from 6- and 24-month old rats was unable to increase caspase-3 enzymatic activity, suggesting that additional factors may be necessary for caspase-3 activation in muscle. Acute insulinopenia stimulates non-lysosomal, ATP-, and proteasome-dependent proteolysis in rats [227]. Subsequent studies identified caspase-3 as the protease likely responsible for proteolyzing actomyosin into the 14-kDa actin fragments observed in the atrophying muscles of diabetic rats [228]. Subsequent studies identify caspase-3 as the protease responsible for proteolyzing actomyosin into the 14-kDa actin fragments observed in the atrophying muscles of diabetic rats [228] and following denervation injury in mice [229]. Importantly, the enzymatic activities of caspases-3, -6, -8, and -9 were found to be significantly increased in the gastrocnemius of cachexic (MAC16 tumor-bearing) as compared to non-cachexic (MAC13 tumor-bearing) mice [230]. Collectively, these studies suggest that increased caspase activity may play a causal role in the muscle atrophy associated with systemic metabolic dysfunction.

Sporadic inclusion body myopathy (sIBM) is the most common degenerative muscle disease in patients aged 50 years or more, and is often referred to as sporadic inclusion-body myositis due to high levels of immune cells, including cytotoxic CD8^+^ T cells, in the endomysium [231]. sIBM patients as a group respond poorly to anti-dysimmune therapies [232] suggesting that immune system dysfunction is likely not causal for sIBM, but rather exacerbates sIBM severity and/or progression. Muscle degeneration in sIBM is characterized by vacuolization and intra-myofiber accumulation of misfolded and polyubiquitinated protein aggregates including amyloid-β (Aβ), SQSTM1, LC3, TDP-43, HSP70, and proteasomal subunits [179,233,234,235,236,237]. In addition, biochemical analyses reveal an increase in LC3-II and SQSTM1 expression along with a decrease in the enzymatic activity of lysosomal enzymes Cathespin B and D despite their increased expression [236]. However, though the increase in LC3-II is indicative of mature autophagosomes, the increase in SQSTM1 would rather suggest a decrease in autophagic flux [238]. Consistent with these observations, transgenic mice overexpressing R155H or A232E VCP mutants commonly observed in humans with IBM associated with Paget’s disease recapitulate the progressive functional and morphological deficits observed in sIBM patients [239]. Treatment of VCP A232E transgenic mice with arimoclomol restored grip strength and maximal EDL tetanic force to near WT levels and markedly reduced ubiquitin and TDP-43 aggregates [240], indicating that dysregulated proteolysis plays a significant role in the pathogenesis of sIBM.

## 4. Unexpected Links

RNA-binding proteins (RBPs) play essential roles in nearly every aspect of cell biology through their direct influence on post-transcriptional gene expression [241,242,243], translational control [244], RNA quality and stability [245], and RNA splicing [246]. A census of human RBPs revealed that ~8% of protein coding genes are directly involved in RNA metabolism (the collective events in the life cycle of RNA molecules including synthesis, secondary structure modulation, base modification/editing, processing, and degradation), either through direct interaction or as essential components of ribonucleoprotein (RNP) complexes [247]. Importantly, alterations in RNA metabolic processes are increasingly being recognized for its contributions to physiological aging and age-associated disease. The roles of RBPs in skeletal muscle typically have been studied in the context of developmental and/or disease processes. Identifying mechanistic links between the activities of RBPs and the cellular processes modulating skeletal muscle physiology in the context of aging is an ongoing pursuit. Here, we will highlight recently identified, and potentially new links, between the activity of RBPs, biological aging, and skeletal muscle.

Altered post-transcriptional gene expression regulation, in part through changes in miRNA expression, play causal roles in the cellular and tissue morphological changes in the cardiovascular systems of the elderly [248,249,250]. SNPs in A-to-I RNA editing genes *ADARB1* and *ADARB2* are associated with longevity in both humans and *Caenorhabditis elegans* [251], and alterations in the editing of the transcriptome is implicated in the etiology of disease [252,253]. In mice, genetic deletion of *Adar* induced lethality around ED11.5 due to severe defects in hematopoiesis and disintegration of the liver [254]. Similarly, mice in which *Adarb2* is genetically deleted develop normally, but die between P0 and P20, becoming progressively seizure prone by P12 [255]. In addition, proteome analyses of 7- and 30-month old rat gastrocnemius muscles indicates a significant increase in the expression of the C-to-U RNA editing enzyme APOBEC2 [256], suggesting that RNA editing in general, and specific subsets in particular (e.g., A-to-I, C-to-U) are temporally regulated in a cell type and context-dependent manner. Interestingly, such regulation may be in response to IIS signaling. ADAR2 expression in pancreatic islets, as well as ADAR2-targeted editing of GluR-B RNA, is positively regulated in obese insulin resistant mice with hyperinsulinemia [257]. A recent study identified ADAR1 T738 (*Adar*) and ADAR2 T553 (*Adarb2*) as being direct targets of Akt kinase activity in cultured human cell lines [258]. Akt inhibition increased, whereas phospho-mimetic mutants of the ADAR1 and ADAR2 Akt sites reduced the RNA editing of select transcripts including *NEIL1*, *CCNI*, *AZIN1*, and *CYFIP2*. Interestingly, millions of RNA editing sites have been identified in human cells [259,260]. However, the vast majority of editing sites in humans and primates are observed in the ubiquitous inverted repeat *Alu* elements largely found in noncoding regions of the genome [260]. Further, and in-depth cDNA sequencing analysis of the brain of a 67-year old male concluded that less than 119 of the transcript sequence variants identified from 541,777 nt of exon sequences analyzed are likely due to post-transcriptional RNA editing [261]. Although these observations would suggest that RNA editing plays a negligible role in introducing post-transcriptional amino acid substitutions in humans, additional studies in young and old individuals are necessary to determine whether RNA editing plays an extensive role in biological aging.

Alterations in RNA processing steps including splicing, poly-adenylation, and 5′ capping) are associated with both physiological aging and age-associated disease [20,262,263,264,265]. It is estimated that >95% of human multi-exon genes are subject to alternative splicing [266]. Brain, heart, and skeletal muscle have the highest levels of evolutionarily conserved alternatively spliced transcripts [267], and age-associated changes in splicing are observed in the transcriptomes of muscles from aged humans [20,268,269] and mice [270]. Importantly, changes in alternative splicing may drive cellular dysfunction through changes in the binding properties, enzymatic activities, and intracellular localization of a large percentage of the proteome [20,262,271], and is implicated as a causal mechanism of cellular aging through its impact on both metabolic processes and DNA repair [272]. In mammals, ~40 proteins and 5 small nuclear RNAs form the core spliceosomal complex [273,274], which is dynamically regulated by the activities of multiple RBPs, including members of the SR protein [275] and heterogeneous nuclear ribonucleoprotein (hnRNP) [276] families. Individual transcript splicing is modulated, in part, by cis-acting elements within the nascent transcript (e.g., exonic splicing enhancers/silencers—ESE/ESS, intronic splicing enhancers/silencers—ISE/ISS) and secondary structure [277]. Importantly, tissue-specific and ubiquitously expressed RBPs cooperatively interact with the regulatory elements both within the nascent transcript and of the spliceosome for context- and tissue-specific mRNA splicing [246,278,279].

Genetic mutations in the splicing factor gene *RBFOX1* have been observed in patients with neurological disorders [280] and in an autistic individual with muscle weakness [281]. Mice in which *Rbfox1* has been specifically deleted in skeletal muscle have decreases in muscle mass and maximal force generation [282], thus establishing a role for Rbfox1 in muscle development and physiology. In addition, mice in which both *Rbfox1* and *Rbfox2* are specifically deleted in adult mice, experienced rapid losses of muscle mass, myofiber cross sectional area, and strength, due in part, to reductions in autophagy and the aberrant expression of calpain-3 [283]. Further, siRNAs targeting *Rbfox1* and/or *Rbfox2* significantly impaired myogenesis in the C2C12 model system through its direct role in promoting the muscle-specific splicing of *Mef2d* [284]. Collectively, these studies establish Rbfox1/2 as essential for muscle developmental processes and the maintenance of muscle mass and strength in adulthood. Whether changes in the expression or activity of these factors contribute to age-associated muscle atrophy is uncertain.

Amyotrophic lateral sclerosis (ALS) is a progressive age-related neurodegenerative disease in which motor neurons are selectively degenerated. Both reductions in nuclear/cytoplasmic ratio of the splicing factor SFPQ, and the aberrant retention of *SFPQ* introns, are observed in motor neurons that were differentiated from ALS patient-derived iPSCs [285], thus suggesting a causal role for SFPQ in the pathogenesis of ALS. Neuron-specific deletion of *Sfpq* in mice led to impaired transcriptional elongation, an effect most pronounced on long genes (>100 kb), and gross developmental abnormalities in the brain [286]. Skeletal muscle-specific deletion of Sfpq similarly induced long gene transcriptopathy and premature death starting around P30 [287]. Between P14 and P30, these mice exhibited significant skeletal muscle growth defects, attributable to the combination of an increase in the percentage of oxidative myofibers, accumulation of excess glycogen, and a decrease in the abundance of OXPHOS complexes I, II, and IV. Although reductions in mitochondrial oxidative capacity are observed in aged and sarcopenic muscles [288,289,290], it is unknown whether dysregulation of SFPQ expression or activity plays a causal role.

A rare cohort of patients have been identified in which heterozygous mutations in *HNRNPU* are associated with intellectual disability and muscle weakness [291,292,293,294]. hnRNP-U is a multifunctional RBP, directly involved in regulating alternative splicing [295,296,297], genome architecture [298], X-chromosome inactivation [299], and is known to promote the DNA base repair through its interaction with NEIL1 [300]. In mice, hnRNP-U protein expression decreases with advancing age in both the heart [301] and skeletal muscle [160], suggesting that loss of hnRNP-U may contribute to age-associated pathologies in these tissues. Mice in which *Hnrnpu* is deleted in striated muscles (*Ckm*-Cre) die around P14 due to a dilated cardiomyopathy (DCM) phenotype, whereas mice in which *Hnrnpu* is deleted specifically within cardiomyocytes (*Myh6*-Cre) die around P10 due to a similar DCM phenotype [301]. Mice in which *Hnrnpu* is deleted specifically in skeletal muscle (*ACTA1*-Cre) are born phenotypically normal, but by 3-months of age develop a myopathy-like phenotype characterized by selective muscle atrophy of glycolytic muscles and histopathological observations of central nuclei, myofiber degeneration, intracellular inclusions, and extensive fibrosis [160]. Changes in the expression and splicing of genes are observed in both cardiac (*Ckm*-Cre) and skeletal muscles (*ACTA1*-Cre). Interestingly, the atrophied skeletal muscles (*ACTA1*-Cre) exhibit signs of metabolic stress. Akt is observed to be constitutive active with its phosphorylation at S473 being desensitized to IGF-1 stimulation, SQSTM1 and Ulk1 phosphorylation at S757 are significantly increased, and multiple genes involved in the transport/import and biosynthesis of amino acids, as well as the repression of oxidative metabolism are significantly reduced in expression [160]. Though high levels of Akt S473 [149] and S6K1 T389 [149] phosphorylation are similarly observed in the atrophied muscles of aged rats, it is unclear whether this is a compensatory effect, or a mechanistic contributor, likely only at specific stages of muscle atrophy.

Splicing of the insulin receptor gene *INSR* is regulated in a cell-type specific context that is dependent upon the development stage or disease condition. Alternative splicing of exon 11 gives rise to protein isoforms INSR-A (exclusion of exon 11) and INSR-B (inclusion of exon 11). INRS-A, which is prevalent during fetal development and in the nervous system, preferentially binds IGF-2 over IGF-1 and insulin, while INRS-B, which is prevalent in adulthood and highly expressed in adipose tissue, liver and skeletal muscle, is more sensitive to insulin [302,303,304]. The splicing of *INSR* is regulated by multiple RBPs including CELF1, SRSF3, MBNL1, and hnRNPs A1, F, and H [305,306,307,308]. Patients with myotonic dystrophy (DM) frequently acquire muscle insulin resistance. Expansion of CTG (DM type 1) and CCTG (DM type 2) repeats in DMPK [309] and ZNF9 [310], respectively, leads to the dysregulation of alternative splicing through MBNL1 loss-of-function and CELF1 gain-of-function [311]. Molecular analyses have identified hnRNP-H, MBNL1, and CELF1 as modulating the increase in the INSR-A:INSR-B ratio observed in DM patients [308,312,313,314]. Although insulin resistance in skeletal muscle is the primary defect in type 2 diabetes [315], and dysregulation of *INSR* splicing occurs in the insulin target tissues of patients with insulin resistance [312,313], the role of *INSR* splicing in the pathogenesis of type 2 diabetes is unclear [303]. Conflicting observations in which the transcript ratio of INSR-A:INSR-B splice variants is either decreased [316] or unchanged [317,318] are reported. Similarly, it has been reported that the INSR-A transcript is exclusively expressed in non-diabetics with the induction of the INSR-B transcript observed in the muscles of type 2 diabetics [319]. To the best of our knowledge, age-associated changes in muscle INSR protein isoform expression, and whether such isoform expression changes are correlated with the development of type 2 diabetes in the elderly is unknown.

Treatment of Ewing sarcoma cells with the ATP-competitive PI3K and mTOR inhibitor Dactolisib induced significant reductions in both cell growth and proliferation [320]. Genes for RBPs with known roles in splicing modulation including *FUS*, *HNRNPM*, *SFPQ*, and *SRSF2* were significantly increased, while coimmunoprecipitation and cosedimentation of hnRNP-M with core spliceosomal proteins were significantly enhanced following treatment. Importantly, siRNA knockdown of *HNRNPM* blocked a subset of Dactolisib induced changes in cassette exon splicing for exons containing proximal hnRNP-M consensus motifs [320]. Interestingly, hnRNP-M expression is positively corelated with SGK1 phosphorylation, and its overexpression rescues the impaired differentiation of C2C12 myoblasts treated with siRNA targeting *Rictor* [321]. Though hnRNP-M physically interacts with Rictor, and siRNAs targeting either *Rictor* or *HNRNPM* repressed insulin-stimulated Akt S473 and SGK1 S422 phosphorylation to a similar degree, the molecular mechanism underlying these observations is unknown. hnRNP-E2 (*PCBP2*) was identified in a two-hybrid screen of a human bone marrow cDNA library with SIN1 (*MAPKAP1*), while siRNAs targeting either SIN1 (*MAPKAP1*) or hnRNP-E2 (*PCBP2*) similarly potentiate an increase in the percentage of apoptotic cells treated with either H_2_O_2_ or TNFα [322]. Though endogenous SIN1 and hnRNP-E2 coimmunoprecipitated in cell lysates and combined siRNA treatments had an additive effect on both H_2_O_2_ and TNFα induced apoptosis, suggesting their participation in a common signaling pathway, the molecular mechanism, presumably activating mTORC2, is unknown. SR protein SF2/ASF (*SFRS1*) positively modulates protein translation through an Akt-independent mechanism [323]. MEF and NIH 3T3 cells overexpressing SF2/ASF had significantly increased S6K1 and 4E-BP1 phosphorylation without increases is Akt phosphorylation at S473. Interestingly, SF2/ASF physically binds to both mTOR and the catalytic subunit of the PP2A phosphatase [324] suggesting that it may facilitate mTORC1-mediated phosphorylation of 4E-BP1 by either recruiting it to the mRNP complex via direct binding of mRNA ESE or perhaps by indirect inhibition of PP2A thereby biasing the phosphorylation-dephosphorylation kinetics of 4E-BP1 toward phosphorylation. Intriguingly, these observations suggest that RBPs involved in nuclear-cytoplasmic shuttling of RNA may positively regulate mTOR-dependent cell survival and growth processes, perhaps as part of a feed forward mechanism to insure selective translation of cell survival and growth transcriptional programs.

## 5. Conclusions

Worldwide, the number of individuals aged 65 years or older is expected to more than double by the year 2050 [325]. In the U.S. alone, it is estimated that approximately 45% of adults greater than 60 years of age have some degree of sarcopenia [326], contributing to the impaired ability to perform activities of daily living and the loss of independence in the elderly population. In contrast with other well-studied diseases, and despite the urgent need by an aging population, muscle atrophy and weakness are frequently considered signs of “normal aging.” Though it is managed by a regimen of resistance training and increased protein intake, only a subset of sarcopenic individuals see improvements in muscle mass, strength, and functionality [11,327]. In the nearly 30 years since the identification of mTOR [328,329,330], the complex and often interrelated signaling pathways regulating skeletal muscle mass, strength, and functionality have begun to be more clearly defined. New mechanisms and linkages continue to be discovered, defining new regulatory circuits while further refining the established pathways. Clearly, there has been much exciting progress in our understanding of these pathways and their contribution to muscle wasting disorders, but much remains to be done.

## Data Availability

Not applicable.

## Abbreviations

**Abbreviation**	**Full name**
Aβ	amyloid-β
ADARB1	adenosine deaminase RNA specific B1
ADARB2	adenosine deaminase RNA specific B2
ADAR	adenosine deaminase RNA specific
APOBEC2	apolipoprotein B mRNA editing enzyme catalytic subunit 2
CAD	carbamoyl-phosphate synthetase 2, aspartate transcarbamoylase, dihydroorotase
FoxO1	Forkhead boxO transcription factor 1
FoxO3	Forkhead boxO transcription factor 3
GAP	GTPase activating protein
GCN2	General control nonderepressible 2 kinase
GH	Growth hormone
GPCR	G-protein-coupled receptor
GSK3	glycogen synthase kinase 3
HRI	Heme-regulated inhibitor kinase
IGF-1	Insulin like growth factor 1
IIS	Insulin/IGF-1 signaling
miRNA	microRNA
MTHFD2	methylenetetrahydrofolate dehydrogenase 2
mTORC1	mTOR containing complex 1
mTORC2	mTOR containing complex 2
PERK	PKR-like ER kinase
PI3K	phosphoinositide 3-kinase
PKR	Protein kinase RNA-activated
PRAS40	proline-rich AKT substrate of 40 kDa
RHEB	Ras homolog enriched in brain
rpS6	ribosomal protein S6
RTK	Receptor tyrosine kinases
S6K1	ribosomal protein S6 kinase 1
sIBM	sporadic inclusion body myopathy/myositis
SNP	single nucleotide polymorphism
ULK1	unc-51 like autophagy activating kinase 1
ULK2	unc-51 like autophagy activating kinase 2
